# Enrichment of High Arsenic Groundwater Controlled by Hydrogeochemical and Physical Processes in the Hetao Basin, China

**DOI:** 10.3390/ijerph192013489

**Published:** 2022-10-18

**Authors:** Wengeng Cao, Yu Ren, Qiuyao Dong, Zeyan Li, Shunyu Xiao

**Affiliations:** 1 Institute of Hydrogeology and Environmental Geology, Chinese Academy of Geological Sciences, Shijiazhuang 050061, China; 2 Key Laboratory of Groundwater Sciences and Engineering, Ministry of Natural Resources, Shijiazhuang 050061, China; 3 Hebei Cangzhou Groundwater and Land Subsidence National Observation and Research Station, Shijiazhuang 050061, China; 4 College of Geosciences and Engineering, North China University of Water Resources and Electric Power, Zhengzhou 450011, China

**Keywords:** high arsenic groundwater, cumulative frequency curve, ionic relationship, hydrogeochemical process

## Abstract

Based on 447 samples collected from a shallow aquifer (depths from 0 to 150 m) in the Hetao Basin, Northern China, an integrated hydrogeochemical approach was used in this study to conceptualize the enrichment of high arsenic groundwater in the Hetao Basin. An unconventional method of distinguishing hydrogeochemical and physical processes from a dataset was tested by investigating the cumulative frequency distribution of ionic ratios expressed on a probability scale. By applying cumulative frequency distribution curves to characterize the distribution of ionic ratios throughout the Hetao Basin, hydrogeochemical indicators were obtained that distinguish the series of hydrogeochemical processes that govern groundwater composition. All hydrogeochemical processes can basically be classified as recharge intensity of groundwater, evaporation concentration intensity, and reductive degree controlling the spatial distribution of arsenic. By considering the three processes, we found that the concentration of arsenic was more than 10 μg/L when the (HCO_3_^−^+CO_3_^2−^)/SO_4_^2−^ ratio was over 4.1 (strong reductive area). As the evaporation concentration intensity increased, the median value of arsenic increased from 10.74 to 382.7 μg/L in the median reductive area and rapidly increased from 89.11 to 461.45 μg/L in the strong reductive area. As the river recharge intensity increased (with the intensity index increasing from 0 to 5), the median value of arsenic dropped from 40.2 to 6.8 μg/L in the median reductive area and decreased more markedly from 219.85 to 23.73 μg/L in the strong reductive area. The results provide a new insight into the mechanism of As enrichment in groundwater.

## 1. Introduction

Arsenic (As) is a toxic element that commonly exists in groundwater. Too much intake of arsenic water can cause skin disease or even cancer in humans [1,2]. The WHO and many countries, including China, have set 10 μg/L as the maximum intake value of arsenic in drinking water due to its harmful impact on human health. Public concerns about arsenic in groundwater have increased in the last 30 years [3,4,5,6,7,8,9,10,11,12]. High arsenic groundwater widely exists in over 100 countries, including the United States, Bangladesh, India, China, etc. Both geological and human causes are key factors contributing to the high As concentration in groundwater. The Hetao Basin is a sediment-filled basin in northern China, and high As groundwater was first reported there in 1994 [13]. Previous studies have shown that the As concentration ranged from 1.1 to 969 μg/L in shallow groundwater, with a significant proportion (up to 90%) of As occurring as As(III) [14]. High As groundwater generally (>50 μg/L) occurs in shallow alluvial–lacustrine aquifers in reducing conditions, and the arsenic in groundwater is believed to have originated from exchangeable As and Fe–Mn oxide-bound As in aquifer sediments in the Hetao Basin [15,16]. The Chinese government has adopted several measures to improve drinking water quality in the Hetao Basin. According to many studies, the high As groundwater in the Hetao Basin is mainly caused by geological, hydrological, hydrogeochemical, and biogeochemical aspects, which means removal of As is challenging [9].

It is essential to determine the hydrogeochemical processes in the aquifer system to quantitatively identify the mechanisms that control As mobilization in groundwater systems [17,18]. Previous studies have shown that hydrogeochemical processes are the main factor controlling the distribution of high arsenic groundwater in the Hetao Basin, Inner Mongolia [19,20]. Many ionic ratios (Cl^−^/Br^−^, SO_4_^2−^/Cl^−^, and Na^+^/Cl^−^) have been jointly used to distinguish hydrogeochemical processes such as redox condition and evaporation concentration [19,21,22,23], identify redox condition, recharge and quantitatively evaluate groundwater systems in arid and semiarid regions [6,11,12,24], and determine the impact of irrigation return flow on arsenic mobilization in groundwater systems [25,26]. Cumulative frequency curves of ionic ratios represent the cumulative process from 0 to 1 with ion increment. When the contents of ion ratios increase significantly, the slope of the cumulative frequency curve will also change correspondingly. Through this approach, the distribution characteristics of ions can be determined by the range of ion aggregation [27,28]. However, the types of ionic ratios used should be distinguished according to the actual hydrogeological environment and climate conditions.

In this study, we used cumulative frequency curves to characterize the distribution of four kinds of ionic ratios throughout the Hetao Basin, which were considered as the hydrogeochemical indicators that represent the appropriate hydrogeochemical processes that govern groundwater composition. The indicators provide reproducible results that define the main processes controlled by the groundwater environment. This study aimed to demonstrate the enrichment mechanisms of As in groundwater, which were identified by various ionic ratios representing hydrogeochemical processes in different parts of the study area.

## 2. Hydrogeological Setting

**Figure 1:**
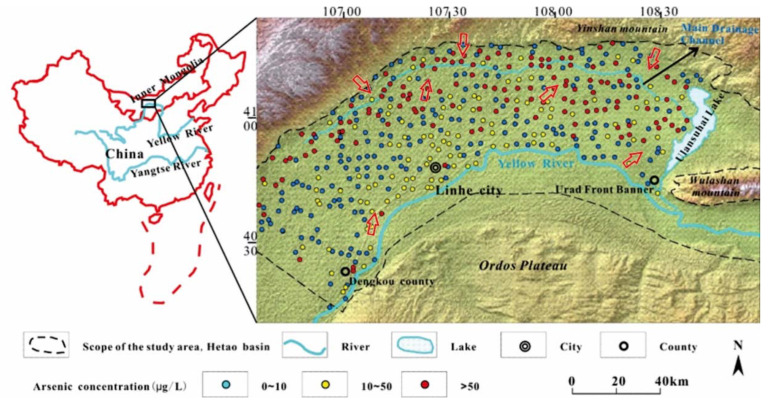
Distribution of groundwater As in the Hetao Plain, Northern China (the red arrow represents flow direction).

The Hetao Basin is a Cenozoic rift basin located in the western part of Inner Mongolia, China, and in the eastern fringe of the Wuranbuh Desert (Figure 1). It is bound by the Yellow River to the south and the Langshan Mountains to the north, with the Ordos Plateau located in the south of the Yellow River. The area is kidney-shaped and covers about 10,000 km^2^. The alluvial basin has a gentle southeastern slope with an elevation between 1060 and 1007 m [19]. The Langshan Mountains are mainly composed of a metamorphic complex (slate, gneiss, and marble), generally of Jurassic to Cretaceous age, which is folded and fractured [29]. The basin is also fault-bound, with a sedimentary environment affected by both paleo-climatic and tectonic events over the past −50 Ma [24,30].

In the Quaternary period, continuous subsidence resulted in the accumulation of thick sedimentary sequences of fine-grained lacustrine sediments during the mid-Cenozoic era. The thickness of the sediment ranges from 500 to 1500 m in the southeast of the basin and from 7000 to 8000 m in the northwest [14].

The average annual precipitation is 169 mm, which is much lower than the evaporation of 2328 mm. The length of the Yellow River mainstream is 345 km, and the annual average transit water runoff can be 31.5 billion m^3^.

The groundwater is recharged by the Yellow River and precipitation, with lateral recharge from the Langshan Mountain in the north. The total drainage channel, located in the piedmont depression of the northern Hetao Basin, is the most prominent drainage area (Figure 1).

## 3. Methods

### 3.1. Field Sampling and Analysis

A total of 447 samples (sampling depths ranging from 0 to 150 m) were collected from the shallow aquifer composed of late Pleistocene and Holocene alluvial and lacustrine deposits. About 80% of samples were from domestic water wells and the rest were from agricultural supply wells. The sampling campaign was conducted during the period from 10 June to 27 September 2013 (Table S2).

The samples used for As speciation analysis were filtered through 0.45 μm membrane filters and stored in new but prerinsed HDPE bottles (Nalgene) at a constant temperature of 4°C. Then, the filtered samples were acidified to pH=1 by the addition of ultrapure HCl. All the samples were sent to the laboratory for further analysis within 5 days. The As speciation were determined in the Laboratory of Hydrogeology and Environmental Geology Institute, Chinese Academy of Geological Sciences (CAGS), with high-performance liquid chromatography (HPLC)–ICP-MS. During the process, the laboratory used an HPLC (1100 Series, Agilent) consisting of a system controller, a solvent delivery module, a column oven, and a 6-port injection valve. A reversed-phase C18 column (Capcell, Pak, 250 mm × 4.6 mm, 5 μm particle size) was adopted to separate As species. An ICP-MS (7500C, Agilent) functioned as a detector in the tests and was operated in the He mode for As determination to remove ArCl interference [11].

### 3.2. Spatial Hydrochemical Analysis Methods

The interrelations of major ions (Na^+^, Ca^2+^, Mg^2+^, K^+^, Cl^−^, HCO_3_^−^, CO_3_^2−^, and SO_4_^2−^) can provide insight into the sources of groundwater and the hydrogeochemical processes leading to their composition. All units are expressed in milliequivalent per liter (meq/L), which converts concentrations to chemical “equivalents”. This contributes to understanding of water–rock processes that may control groundwater composition. Because of the log-normal distribution of data, all axes are presented on a logarithmic scale. According to the ion ratios, secondary processes within the total sample group can be identified by assessing good statistical correlations.

To test this supposition, a selection of ionic ratios was assessed with cumulative frequency distribution curves to identify hydrogeochemical and physical processes. Based on ionic relationships, water types could be characterized using the demarcation values established. Inflection points on each graph represented each group’s demarcation values. Cumulative frequency plots of chemical constituents are useful to distinguish background and ambient values as well as values affected by certain factors such as seawater intrusion or anthropogenic contamination [18,31,32]. When the cumulative frequency increases, the ratio grows and the number of samples increases. The slope of curves would change significantly due to the different concentrated distribution range of the data. Hence, the curve was divided into various sections based on the different slopes. The results of these tests are presented in figures below, including the geographical distribution of the established groups and their position on Piper diagrams.

## 4. Result

### 4.1. Spatial Distribution of Arsenic

Among the results of shallow groundwater samples, 53.5% had As concentration exceeding 10 μg/L in the Hetao Basin, while 134 of them contained more than 50 μg/L, accounting for 30.0% of all samples (Figure 1). Two arsenic anomaly belts were found. The first one was between the piedmont alluvial–proluvial plain and the Yellow River alluvial–lacustrine plain along the total drainage channel. This district is the groundwater discharge area of the Hetao Basin, where the groundwater is of poor quality and with high pH, TDS (total dissolved solids), Cl^−^, and HCO_3_^−^ and low SO_4_^2−^. The second place was distributed in alluvial fans of the Yellow River along the north bank. Arsenic content in this region were at a relative low level ranging from 10 to 50 μg/L compared to that in the former place.

Most high arsenic samples were collected at depths of 30 m, illustrating that shallower aquifer promotes enrichment of arsenic (Figure S1a). Moreover, various arsenic species were found in different redox environments. As^3+^ was usually found in the reducing environment and As^5+^ in the oxidation environment. Samples with As^3+^/As^5+^ > 1 were mainly distributed at depths within 30 m (Figure S1b).

### 4.2. Hydrogeochemical Process Based on Ionic Ratios

#### 4.2.1. Na^+^/Cl^−^ Groups

**Figure 2:**
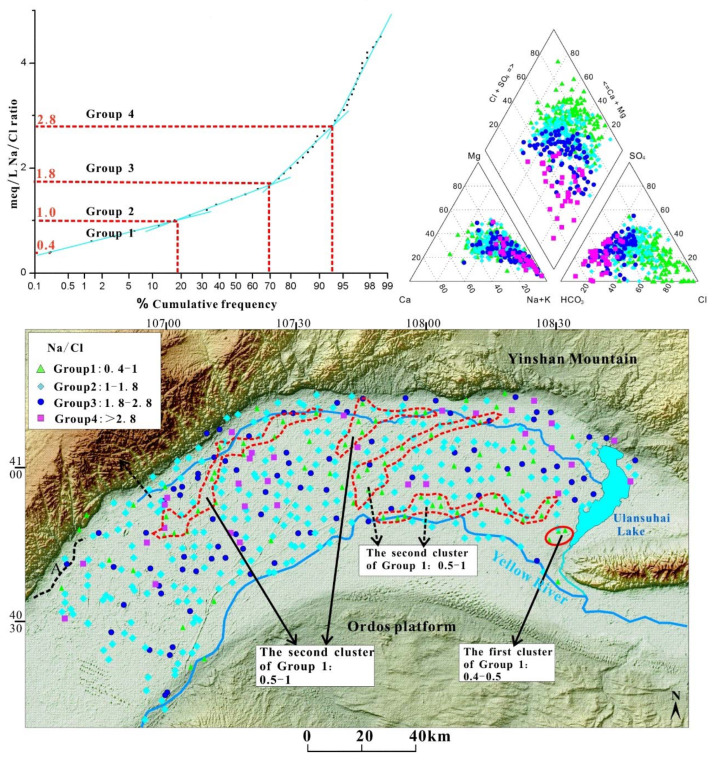
Distribution of Na^+^/Cl^−^ groups in the Hetao Basin from cumulative frequency distribution curve and Piper diagram.

According to the cumulative frequency plot of Na^+^/Cl^−^ (Figure 2), four possible groups could be identified based on different slopes of curve segments. The interval was not classified as a group between ratios of 0 to 0.4 as no samples were found in this range. The threshold of groups for each segment of Na^+^/Cl^−^ cumulative frequency curve could be considered subjective due to slight perturbation in each segment, and the interpretation of each group might be considered empirical as a result. The significant change of the ionic ratios ranges revealed it could present the hydrology characteristics of the aquifer.

The upper limit of Group 1 samples was around 1, representing all samples in Group 1 with Cl^−^ excessing Na^+^. This group covered nearly 20% of the total, and two separate clusters could be formed on the basis of the sample distribution of the Piper diagram (Figure 2). The first cluster was similar to the brine samples, while the second cluster formed an obvious range at the top of the diamond field. From the perspective of spatial distribution, the samples in the first cluster were collected from the northern slope of the Wulashan Mountain and the west of Ulansuhai Nur of west Hetao Basin (refer to the solid red line). The second cluster samples were the discharged groundwater in the central part of the Hetao Basin around the drainage channels.

Unlike other groups in the Na^+^/Cl^−^ plot, the straight line segment defining Group 2 (ratio: 1–1.8) indicated that samples of Group 2 were distributed normally. The Piper diagram showed that samples from this group were not only spread across the diamond field but also in each ternary field. In terms of spatial distribution, the samples were widely distributed throughout the studied area where fresher groundwater could often be found. Accounting for 57.2% of the total, this group could be considered the “background” group representing typical arid and semiarid inland basin groundwater.

Group 3 (ratio: 1.8–2.8) and Group 4 (ratio: >2.8) were not distributed as widely as Group 2 (together representing about 25.6% of the whole dataset) and tended to reflect the dominance of HCO_3_^−^ over Cl^−^ rather than an increase in Na^+^ on the Piper diagram. This might have resulted from the prevalence of HCO_3_^−^ in the groundwater without the effects of Cl^−^ input. These two groups indicated that excess Na^+^ was present compared to Cl^−^ due to mineral hydrolysis or ion-exchange reaction.

#### 4.2.2. (HCO_3_^−^+CO_3_^2−^)/Cl^−^ Groups

The curves of Na^+^/Cl^−^ (particularly Groups 3 and 4) demonstrated that variations were controlled mostly by the relationship between Cl^−^ and HCO_3_^−^ rather than the relative proportion of Na^+^ to other cations. A plot of HCO_3_^−^ and CO_3_^2−^ versus Cl^−^ was drawn to assess the distribution of this ratio and was divided into three groups as shown in Figure S2.

Group 1 (0–1.3) included the most number of samples of all data ranges, with samples extensively distributed in the Hetao Basin, and accounted for 62.7% of all samples. The samples were mainly distributed in the alluvial–lacustrine plain of the Yellow River. Samples in Group 1 were Cl^−^ dominant, while samples in Groups 2 and 3 were HCO_3_^−^ dominant. Geographically, the samples were mainly distributed among the alluvial fan at the north, the Yellow River, and the canals, which were all weak runoff areas. Meanwhile, the groundwater there had the chemical feature of salt evaporation.

Samples in Groups 2 (ratio: 1.3–2.4) and 3 (ratio: >2.4) accounted for 25.6% and 11.7% of all data, respectively. These two groups had similar hydrogeochemical characteristics and were mostly restricted to the left side of the diamond field where HCO_3_^−^ was prevailing over Cl^−^. Most of the samples were taken from the fresher inland groundwater of the main aquifer. The main hydrochemical type of groundwater was Na-Mg-Ca-HCO_3_. In terms of spatial distribution, samples in Groups 2 and 3 were distributed in similar areas. These samples could be divided into two clusters. The samples in the first cluster of Groups 2 and 3 were mainly distributed in the piedmont alluvium and diluvium plain on the south side of the Langshan and Seertengshan Mountains. To be more specific, the samples in Group 2 were located on the alluvial and diluvial fans and were scattered in the depressions in front of the fans and depressions between the fans in Group 3. The samples in the second cluster were especially concentrated along the main canals of the Yellow River.

#### 4.2.3. Ca^2+^/(HCO_3_^−^+CO_3_^2−^) Groups

The curves of (HCO_3_^−^+CO_3_^2−^)/Cl^−^ and Na^+^/Cl^−^ indirectly supported the association of HCO_3_^−^ and Ca^2+^ when occurring at the same time. Accordingly, the ratio of Ca^2+^/(HCO_3_^−^+CO_3_^2−^) revealed a strong relationship between the two ions. The cumulative frequency curve was divided into four groups (Figure S3).

Group 1 (0–0.15) samples comprised 9.7% of all samples and typically represented the condition of high HCO_3_^−^ compared to high Cl^−^. On the cation and anion ternary field, the samples represented groundwater with high Na^+^ and low Ca^2+^, Mg^2+^, and SO_4_^2−^. It was also found that the main hydrochemical types in the groundwater in Group 1 were Na-HCO_3_ and Na-HCO_3_-Cl. Geographically, as shown in Figure S3, Group 1 samples were mainly distributed in the lowest-lying areas of the Hetao Basin center (the dotted red line in Figure S3). Group 1 groundwater existed in an environment with the feature of strong reduction, evaporation and concentration, and ion-exchange reaction intensity.

Samples in Groups 3 (0.6–1.2) and 4(>1.2) reflected more Cl^−^ on the top position of the Piper diamond field, while HCO_3_^−^ was dominant over Cl^−^. Geographically, according to the distribution of the ratio range, two separate clusters could be formed in the Hetao Basin. The first cluster of Groups 3 and 4 was located in the piedmont of the Yinshan Mountain, the north margin of the Hetao Basin, and were consequently associated with fresher groundwater either because of recharging Ca-HCO_3_ water or (more likely) due to the effect of the by-product of mineral hydrolysis of Ca feldspars. The samples in the second cluster were distributed along the side of the Yellow River and among the canal system irrigation areas in the center of the Hetao Basin, representing the effect of the Yellow River recharge. In this cluster, Group 4 samples were mainly distributed in the depression among the alluvial–diluvial fans or interfluvial lowlands, representing ratios great than 1, or Ca-HCO_3_ type water where excessive Ca was derived from another source.

Group 2 (0.15–0.6) accounted for about 44.4% of all samples, which was the largest proportion among all the groups. On the Piper diamond field, groundwater in Group 2 was located between samples in Groups 1 and 3. The samples represented the intermediate conditions in the hydrogeochemical process from Group 3 groundwater to Group 1.

#### 4.2.4. (HCO_3_^−^+CO_3_^2−^)/SO_4_^2−^ Groups

Four groups could be easily identified from the cumulative distribution curve of total carbonate (HCO_3_^−^+CO_3_^2−^) with SO_4_^2−^ (Figure S4). The predominant groups were Groups 1 (0–1.4) and 2 (1.4–2.6), representing 53% and 42.8% of the total dataset, respectively. Samples in Group 3 (2.5–4.1) comprised 17.9%, while those in Group 4 (>4.1) represented only 8.8%.

Differentiation of these groups on the Piper diagram did not reveal any unusual or unexpected groupings (Figure S4). Samples in the anion ternary field were typically distributed between the HCO_3_^−^ and SO_4_^2−^ end-members of the Piper diamond field.

Samples in Group 1 were widely spread and mostly confined in the recharge area of the Yellow River and canals (the green point in Figure S4). With the highest relative ratio of SO_4_^2−^ to (HCO_3_^−^+CO_3_^2−^), these samples represented a weak reductive environment. Samples in Group 4 were limited to the lowest place in the Hetao Basin. Different from other groups, the highest ratio representing these samples mainly existed in the strongest reductive environment. Samples in Groups 2 and 3 could be regarded as a transition between groundwater in Groups 1 and 4. (HCO_3_^−^+CO_3_^2−^)/SO_4_^2−^ ratio is an important sensitive factor reflecting redox conditions due to the distinct zoning characteristic.

#### 4.2.5. Others

(1) Na^+^/HCO_3_^−^: Four groups could be identified in the cumulative frequency distribution curve of Na^+^/HCO_3_^−^ (Figure S5), but the general trend of the plot was upward-curving with no significant inflection points demarcating different groups. Therefore, the four group intervals defined here may not be accurate but represent general changes in the gradient of each segment of the curve.

With dominant Na^+^/HCO_3_^−^ ratio distributions, Groups 1 (0.4–1) and 2 (1–2.3) accounted for 30% and 42.8% of all samples, respectively, while Groups 3 (2.3–3.8) and 4 (>3.8) only accounted for 17.9% and 9.3%, respectively. On the Piper diagram (Figure S5), these groups were sequentially distributed across the diamond field from left to right as the proportion of HCO_3_^−^ to Na^+^ changed.

Widely spread and mostly distributed near the Yellow River irrigation areas and the piedmont alluvial–diluvial fans, Group 1 samples represented a zone influenced by the Yellow River and canals due to HCO_3_ type water recharged by freshwater, precipitation, and the lateral flow from the Yinshan Mountain. Group 4 samples were mainly distributed in the paleo-lake, which was the zone of swamping and depression in the Yellow River, representing halite dissolution or deep fracture brine intrusion processes [19]. Groups 2 and 3 samples represented the intermediate conditions in the hydrogeochemical process from Group 4 groundwater to Group 1.

Unlike other ionic ratios, samples plotted along a Cl^−^/SO_4_^2−^ cumulative frequency graph did not show clear inflection points to help define groups of this ratio (Figure S6). Instead, the slope of the curve did not change significantly even though the curve of Cl^−^/SO_4_^2−^ still showed five distinguished groups.

A total of 41.3% of samples fell into Group 1 (0–1), forming two geographically separate clusters in the Hetao Basin (the green point in Figure S6). The first cluster was distributed in the alluvial–diluvial fans along the foot of the Yinshan Mountain. The groundwater runoff intensity of the Group 1 area was high, and people have previously found several large ore deposits of polymetallic sulfide in the Yinshan Mountain, which have been mined on a big scale over many years. SO_4_^2−^ mainly came from the lixiviation of polymetallic sulfide deposits. The second cluster samples were distributed across the Yellow River irrigation area along the canals. The groundwater there was influenced by irrigation with Ca-HCO_3_ type water from the Yellow River. Group 1 samples were from the groundwater of the recharge area, which renewed faster than the other groups and was susceptible to external influences.

(2) Cl^−^/SO_4_^2−^: Groups 2 (1–2) and 3 (2–3.3) samples accounted for 30% and 13.7% of samples, respectively, and were evenly distributed between the north recharge area of the piedmont alluvial–diluvial fans and the south recharge area of the Yellow River. From the Piper diagram, it was easy to see that samples of these two groups showed an increase in Na and a decrease in Mg and Ca. The two groups showed that the samples were collected from the runoff area with an increase in the TDS value and reduction degree.

Groundwater in Groups 4 (3.3–6) and 5 (>6) was controlled by the association of the dominant Cl^−^ ion with the major cation. The presence of SO_4_^2−^ might be insignificant in the classification of these two groups. On the cation triangle of the Piper diagram, the groups also showed a higher Mg^2+^ to Ca^2+^ ratio. The proportion of SO_4_^2−^ in Group 4 was the lowest among the four groups, and the local groundwater represented desulfation. From the perspective of renewability, the groundwater of Group 5 indicated a high-salt and strong reduction environment because of the relatively enclosed environment. Group 4 samples were more renewable than Group 5 samples. In terms of the distributed location, Group 5 samples were concentrated in several depressions along the total drainage canal in the Hetao Basin center, representing a strong evaporation and concentration condition.

## 5. Discussion

### 5.1. Hydrogeochemical Indicators Based on Ionic Ratios

The analysis of cumulative frequency distribution curves has been proven to be a simple but valuable tool for characterizing the distribution of ionic ratios throughout the Hetao Basin. This study showed that variations in ionic relationships could be different by assessing their cumulative frequency distribution (Figure 2 and Figures S2–S6). The success of this approach was dependent on different ion ratios, but it was mostly possible to assign hydrogeochemical and physical indicators that distinguish the key hydrogeochemical and physical processes that govern groundwater composition.

Na^+^/Cl^−^ ratio is a critical indicator representing the salt rock dissolution effect or ion-exchange processes. An increase in relative Na^+^ content represents strong salt rock dissolution or ion-exchange effects with an increase in the ratio. With the lateral or river recharge increasing, the relative Ca^2+^ content increases and the Na^+^ relative content shows an opposite trend. Meanwhile, with increasing reductive environment, the organic matter can transform to (HCO_3_^−^+CO_3_^−^) as the electron donor, while the SO_4_^2−^ content decreases due to it turning into S^2−^ [33]. The key indicators are defined in Table 1 and could illustrate some common characteristics between various ratios.

### 5.2. Hydrogeochemical Zonation

Each hydrogeochemical or physical process from Table S1 can basically be classified as recharge intensity of groundwater, evaporation concentration intensity, and reductive degree.

River recharge and the piedmont lateral recharge were distinguishable using the ionic ratios and the rating standards from Table 1. The recharge intensity of groundwater increased progressively with the grading index ranging from 0 to 5. We defined the samples as 5 if it met the five ionic ratios (Table 2), representing strong recharge area. We defined the sample as 4 if it met four of the five ionic ratios, etc. Zero represented samples that did not meet any of the five ionic ratios, meaning that the groundwater sample obtained no supply from the Yellow River or Yinshan Mountain. The samples whose indicator ranged from 1 to 5 represented groundwater with high HCO_3_^−^ and low Cl^−^ and Na^+^. The main hydrochemical types were Ca-HCO_3_ and Ca-HCO_3_-SO_4_. Geographically, the strong recharge area was divided into two clusters. The first cluster was mainly distributed in the alluvial-diluvial fans along the foot of the Yinshan Mountain, while the second cluster was distributed in the alluvial–lacustrine plain of the Yellow River along the Yellow River and several irrigation channels.

The degree of evaporation concentration can be rated with the index number 0 to 3. Such grading method is similar to that of the recharge intensity (Table 2). The main hydrochemical type of samples with an index ranging from 1–3 are Na-Cl-HCO_3_ or Na-Cl type.

**Figure 3:**
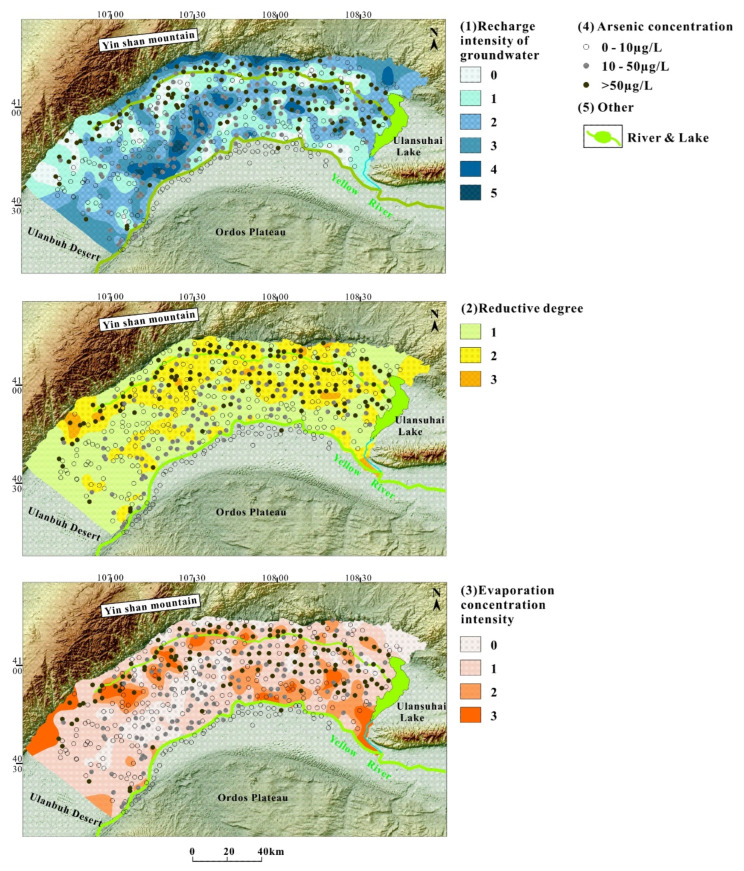
The regionalization map of the hydrogeochemical and physical processes.

The reductive degree can be rated with the number 1 to 3, reflecting increasing levels of reduction (Table 2). (HCO_3_^−^+CO_3_^2−^)/SO_4_^2−^ ratio reflects the reduction degree of the groundwater environment, and the median reductive range is 1.4–4.1. If the value is less than 1.4, it will be considered as weak reductive environment, while it will be considered as strong reductive environment if the value is more than 4.1 (Figure 3).

### 5.3. Hydrogeochemical Control on As Distribution

**Figure 4:**
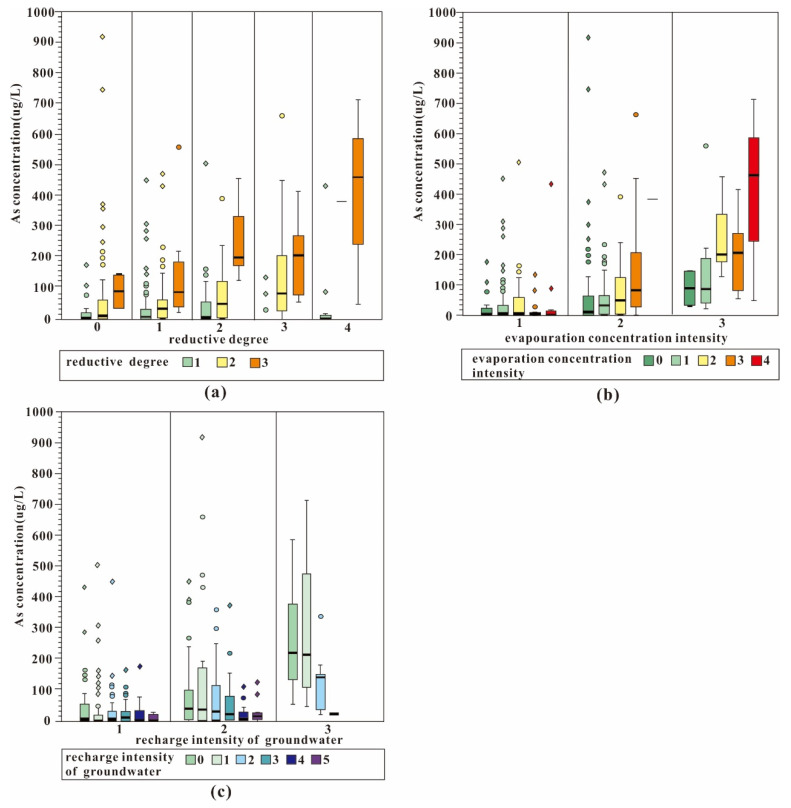
The compound box-plot of arsenic with evaporation concentration intensity, reductive degree, and recharge intensity in the Hetao Basin.

The factors controlling the high arsenic groundwater in the Hetao Basin are very complex. Distribution characteristics cannot be simply explained with a single hydrogeochemical or physical process. As previously mentioned, this study used compound box-plots (Figure 4) to analyze and compare the characteristics of arsenic in shallow groundwater under hydrogeochemical processes by jointly considering the evaporation concentration effect, reductive condition, and recharge intensity.

From Figure 4a, the arsenic concentration in shallow groundwater increased with the increase of the reductive degree (from 1 to 3) in the Hetao Basin. Arsenic which adsorbed to the Fe/Mn-oxides could release to the groundwater due to Fe-oxide reductive dissolution. Among them, the reductive degree with the index equal to 1 was defined as weak reductive condition, with only 10.2% of the selected samples exceeding 10 μg/L. The exceeding proportion of the median reductive area (reductive degree of 2 from Figure 4a) was 59.5%. The exceeding rate of the strong reductive area where (HCO_3_^−^+CO_3_^2−^)/SO_4_^2−^ > 4.1, or reductive degree 3, was up to 100% (Figure 4a).

The evaporation and concentration effect also plays an important role in the migration and enrichment of arsenic in shallow aquifers [19]. Arsenic content increased gradually as the evaporation concentration intensity increased in different reductive partitions. As the evaporation concentration intensity increased from 0 to 4, the median value of arsenic increased from 10.74 to 382.7 μg/L in the median reductive area and from 89.11 to 461.45 μg/L in the strong reductive area (Figure 4b). The shallow water depths in the study area means the strong evaporation effect happen which intensify the increment of the arsenic concentration.

Groundwater was mainly recharged from irrigation of the Yellow River in the Hetao Basin. About 5.2 billion m^3^ of water is diverted each year from the Yellow River to irrigate this basin [34]. As the river recharge intensity increased (with the intensity index increasing from 0 to 5), the median value of arsenic dropped from 40.2 L to 6.8 μg/L in the median reductive area and decreased markedly from 219.85 to 23.73 μg/L in the strong reductive area (Figure 4c). The recharge from the surface water or lateral could introduce more oxygen to the groundwater causing the less reductive environment which is not conducive to the arsenic releasing [28,35].

## 6. Conclusions

This study used cumulative frequency curves of four kinds of ionic ratios to distinguish the different mechanisms of arsenic enrichment in various areas of the Hetao Basin. This approach is unique as it does not require any underlying assumptions and uses natural statistical separations to define water types rather than describing the water type based on observed spatial distributions. Based on the results, we can draw the following conclusions.

(1) About 53.5% of shallow groundwater samples had As concentration exceeding 10 μg/L in the Hetao Basin. There were two arsenic anomaly belts: the first was located in the connected zone between the two main geomorphic units of the northern Hetao Basin along the total drainage channel, namely, the piedmont alluvial–proluvial plain and the Yellow River alluvial–lacustrine plain. The second was distributed in the several large crevasse splay from Dengkou country to Urad Front Banner along the north bank of the Yellow River. Compared to the first high arsenic belt, the arsenic content of this region was at a relative low level with a range of 10–50 μg/L.

(2) Cumulative frequency distribution curves of various ionic ratios, including Na^+^/Cl^−^, (HCO_3_^−^+CO_3_^2−^)/Cl^−^, Ca^2+^/(HCO_3_^−^+CO_3_^2−^), Na^+^/HCO_3_^−^, Cl^−^/SO_4_^2−^, and (HCO_3_^−^+CO_3_^2−^)/SO_4_^2−^ could identify three main factors: reductive degree, recharge intensity, and evaporation concentration intensity. Overlay analysis of these three main processes of high As groundwater could be used to constrain the hydrogeochemical processes that control As mobilization and enrichment in groundwater.

(3) The reductive degree of the Hetao Basin aquifer is believed to be the most important factor controlling the spatial distribution of arsenic. The arsenic concentration in shallow groundwater increased with the reductive degree increasing as represented by (HCO_3_^−^+CO_3_^2−^)/SO_4_^2^. The evaporation and concentration effect accelerated the enrichment of arsenic in the shallow groundwater, while the recharge from the Yellow River and piedmont lateral played the opposite role.

## Figures and Tables

**Table 1:** Hydrogeochemical indicators for various chemical and physical processes controlling groundwater composition in the Hetao Basin based on analysis of cumulative frequency distribution curves.

Ionic Ratio	Hydrogeochemical and Physical Indicator Range (meq/L)	Hydrogeochemical and Physical Processes
Na^+^/Cl^−^	0–1	Saline groundwater, evaporative concentration of NaCl
	1–1.8	Background
	>1.8	Mineral hydrolysis or ion-exchange reaction
(HCO_3_^−^+CO_3_^2−^)/Cl^−^	0–1.3	Weak runoff area, evaporation concentration of salt
	>1.3	River recharge, fresher groundwater
Ca^2+^/(HCO_3_^−^+CO_3_^2−^)	0–0.15	Strong reducing, evaporation concentration
	0.15–0.6	Intermediate process
	>0.6	Recharge by Ca-HCO_3_ water (river recharge), fresher groundwater
Na^+^/HCO_3_^−^	0.4–1	Irrigation of the Yellow River
	1.1–3.8	Intermediate condition
	>3.8	Halite dissolution or deep fracture brine intrusion
Cl^−^/SO_4_^2−^	0–1	Lateral flow recharge from the Yinshan Mountain or the Yellow River, renew faster
	>3.3	High-salt, strong reduction, evaporation concentration
(HCO_3_^−^+CO_3_^2−^)/SO_4_^2−^	0–1.4	Weak reduction
	1.4–4.1	Intermediate condition
	>4.1	Strong reduction, discharge area with high TDS

**Table 2:** The rating standard of the hydrogeochemical process.

Hydrogeochemical and Physical Process	Ionic Ratio	Classification Methodology
**Recharge intensity of groundwater** (from Yellow River recharge or lateral flow recharge from the Yinshan Mountain (fresher groundwater, renew faster))	(HCO_3_^−^+CO_3_^2−^)/Cl^−^: >1.3	Rate the recharge intensity on a scale of 0 to 5 to reflect increasing levels of groundwater recharge intensity from the Yellow River. We defined the samples as 5 if they met five ionic ratios and 0 if they did not meet any of the ionic ratios, etc.
	Ca^2+^/(HCO_3_^−^+CO_3_^2−^): >0.6	
	Na^+^/HCO_3_^−^: 0.4–1	
	Cl^−^/SO_4_^2−^: 0–1	
	Na^+^/SO_4_^2−^: 0.8–1.6	
**Evaporative concentration** (saline groundwater, high salt)	Na^+^/Cl^−^: 0–1	Rate the evaporation concentration on a scale of 0 to 3 to reflect increasing levels of evaporation concentration intensity from the Yellow River. We defined the samples as 3 if they met three ionic ratios and 0 if they did not meet any of the ionic ratio, etc.
	(HCO_3_^−^+CO_3_^2−^)/Cl^−^: 0–1.3	
	Cl^−^/HCO_3_^−^: >3.3	
**Reductive condition**	(HCO_3_^−^+CO_3_^2−^)/SO_4_^2−^: 0–1.4	Weak reduction, defined as 1
	(HCO_3_^−^+CO_3_^2−^)/SO_4_^2−^: 1.4–4.1	Median reduction, defined as 2
	(HCO_3_^−^+CO_3_^2−^)/SO_4_^2−^: >4.1	Strong reduction, defined as 3

## Data Availability

The data associated with the study are not publicly available but are available from the corresponding author upon reasonable request.

## Supplementary Materials

The following supporting information can be downloaded at: https://www.mdpi.com/article/10.3390/ijerph192013489/s1, Figure S1: Plot of arsenic distribution at different sampling depths (a: Total arsenic content at different sampling depths; b: Arsenic species distribution at different sampling depths); Figure S2: Distribution of (HCO_3_+CO_3_)/Cl groups in the Hetao Basin from cumulative frequency distribution curve and Piper diagram; Figure S3: Distribution of Ca/(HCO_3_+CO_3_) groups in the Hetao Basin from cumulative frequency distribution curve and Piper diagram; Figure S4: Distribution of (HCO_3_+CO_3_)/SO_4_ groups in the Hetao Basin from cumulative frequency distribution curve and Piper diagram; Figure S5: Distribution of Na/HCO_3_ groups in the Hetao Basin from cumulative frequency distribution curve and Piper diagram; Figure S6: Distribution of Cl/SO_4_ groups in the Hetao Basin from cumulative frequency distribution curve and Piper diagram; Table S1: Summary of hydrochemical feature of the shallow groundwater in the Hetao Basin; Table S2: Information on the groundwater samples.
