# Supplementary material for: Enrichment of High Arsenic Groundwater Controlled by Hydrogeochemical and Physical Processes in the Hetao Basin, China
Source: Int J Environ Res Public Health. 2022 Oct 18;19(20):13489. doi: 10.3390/ijerph192013489 (PMC9658607; doi:10.3390/ijerph192013489)
Supplement: Supplementary file 1 [file ijerph-19-13489-s001.zip › ijerph-1944010-supplementary.pdf]

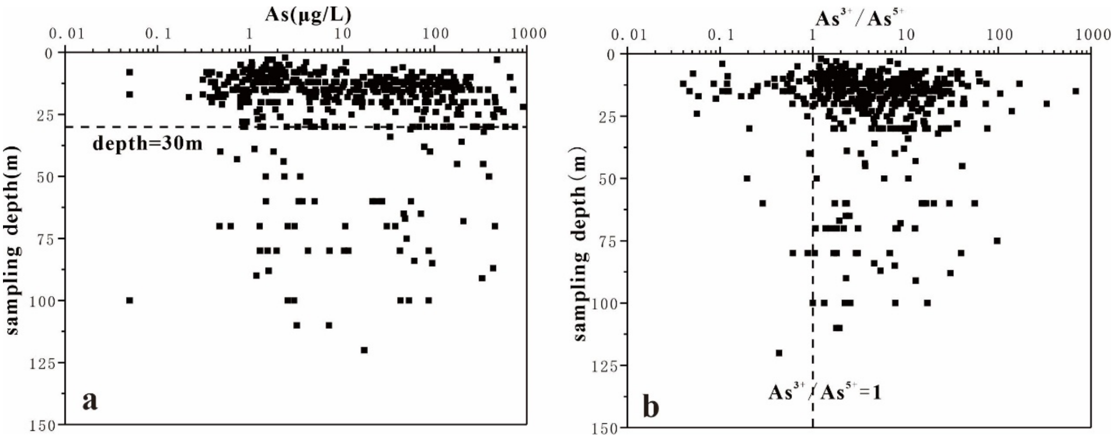

Figure S1. Plot of arsenic distribution at different sampling depths (**a**): Total arsenic content at different sampling depths; (**b**): Arsenic species distribution at different sampling depths）.

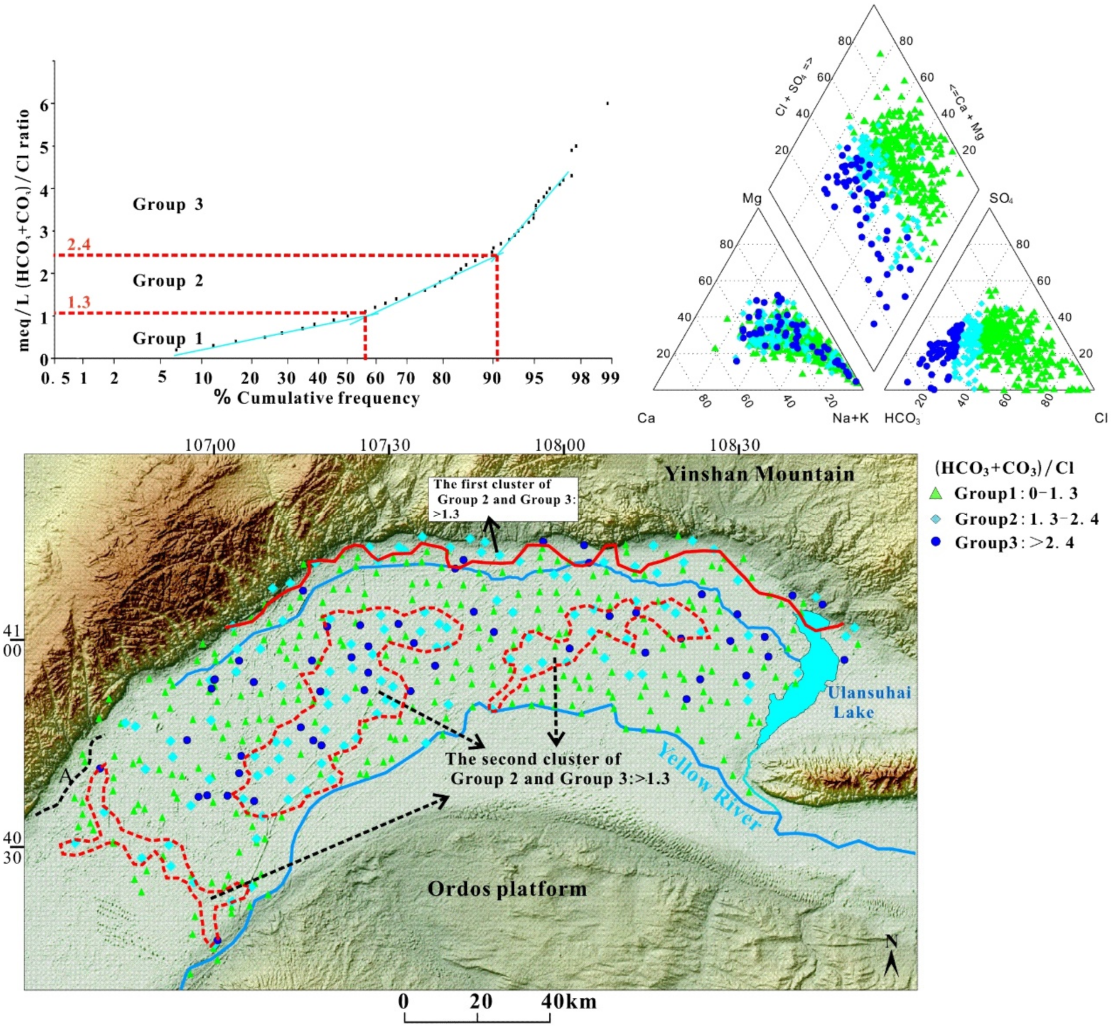

Figure S2. Distribution of $(HCO_3^- + CO_3^{2-})/Cl^-$ groups in Hetao basin from cumulative frequency distribution curve and piper diagram.

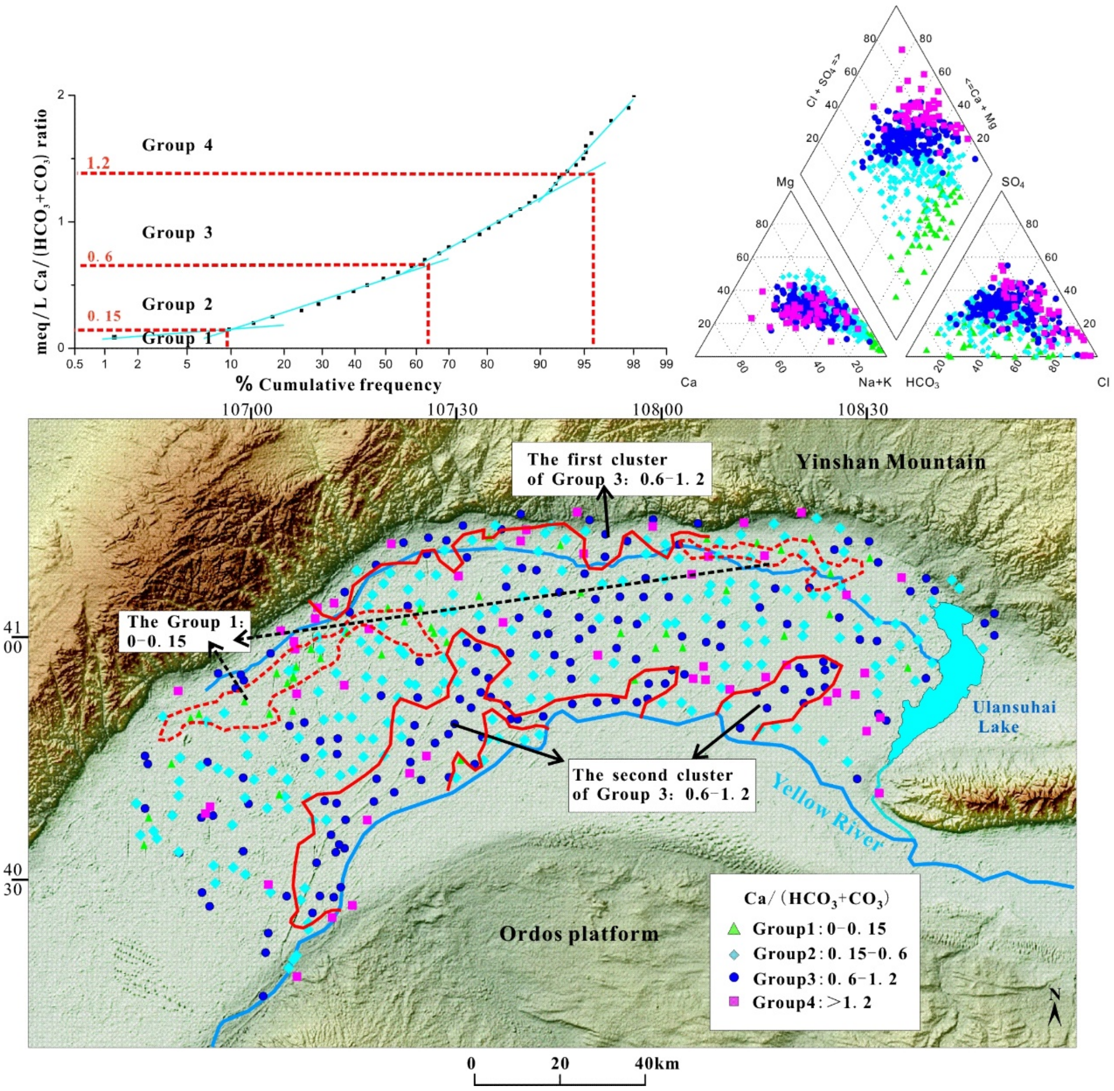

Figure S3. Distribution of $Ca^{2+}/(HCO_3^{-}+CO_3^{2-})$ groups in Hetao basin from cumulative frequency distribution curve and piper diagram.

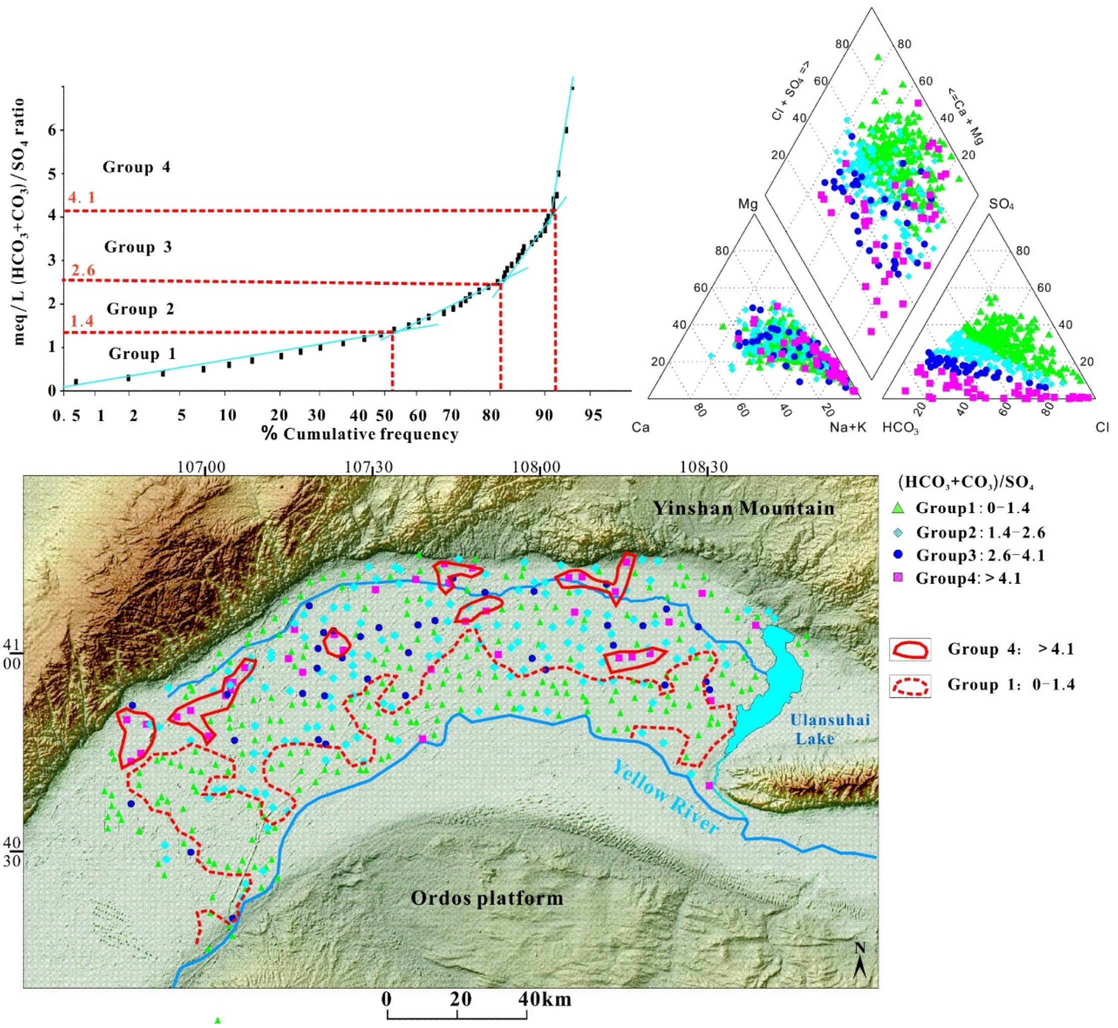

Figure S4. Distribution of $(HCO_3^-+CO_3^{2-})/SO_4^{2-}$ groups in Hetao basin from cumulative frequency distribution curve and piper diagram.

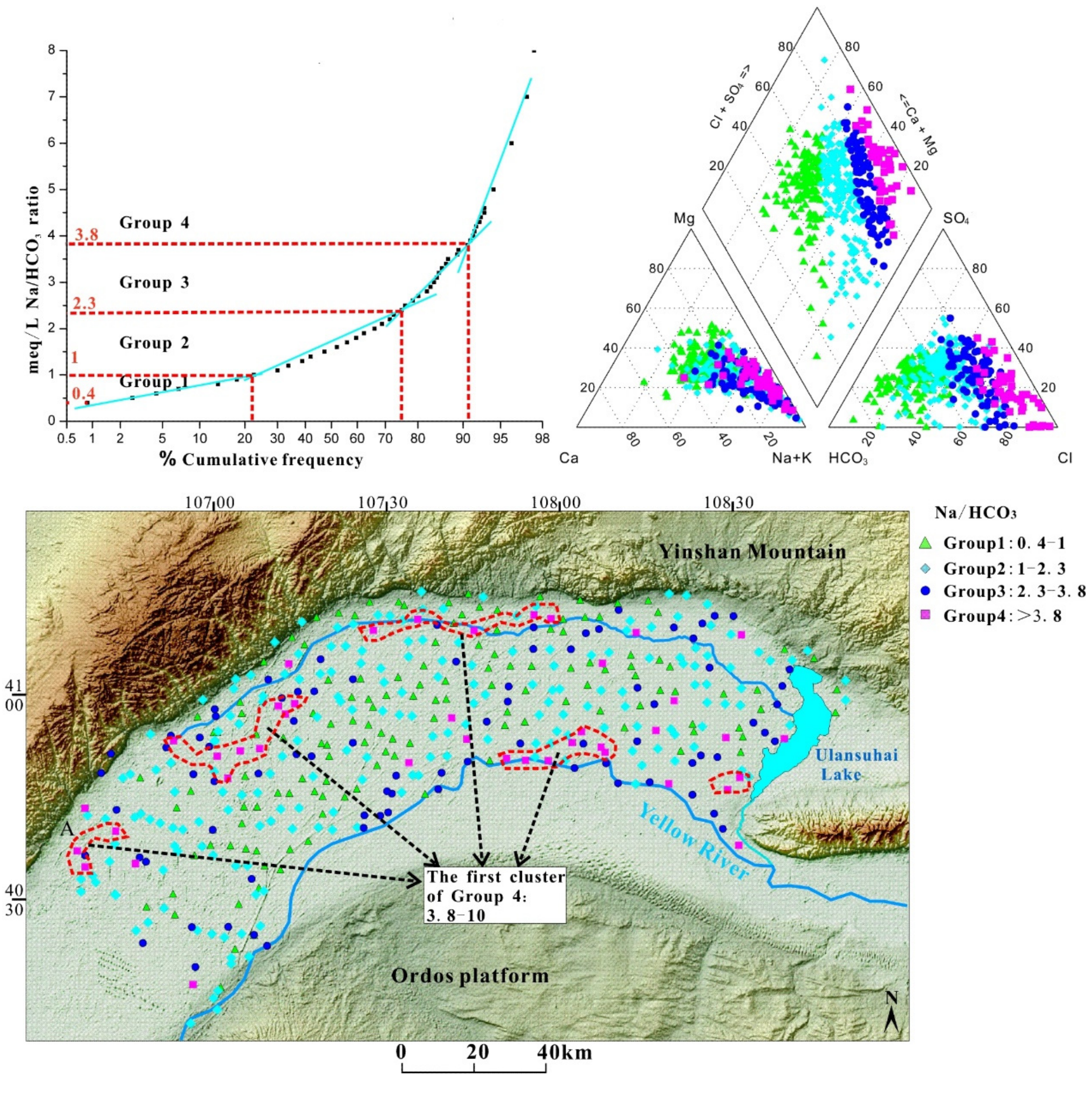

Figure S5. Distribution of Na^+/HCO_3^- groups in Hetao basin from cumulative frequency distribution curve and piper diagram.

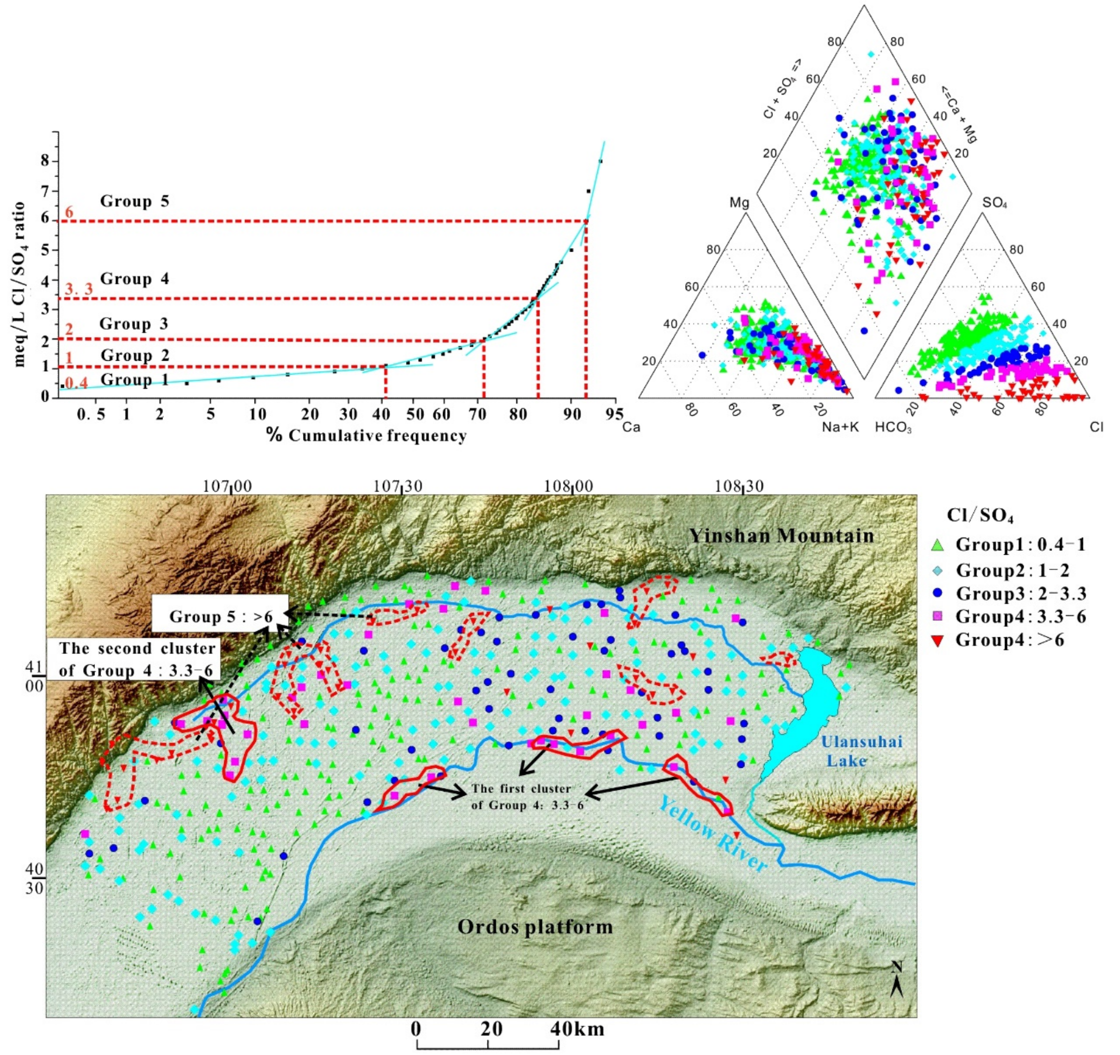

Figure S6. Distribution of Cl^{-}/SO_4^{2-} groups in Hetao basin from cumulative frequency distribution curve and piper diagram.

Table S1. Summary of hydrochemical feature of the shallow groundwater in the Hetao basin.

element	Unit	average	median	standard deviation	range	min.	max.	percentile %		
								25	50	75
As	μg/L	63.82	14.24	120.4	916.7	0	916.7	1.84	14.24	65.19
As^{3+}	μg/L	54.64	10.17	104.79	719.4	0	719.4	0.9	10.17	55.77
As^{5+}	μg/L	9.18	1.54	20.46	224.6	0	224.6	0.46	1.54	8.19
Fe	mg/L	1.99	0.84	3.72	55	0	55	0.18	0.84	2.4
Na^{+}	mg/L	441.93	280.8	581.76	6369.02	35.98	6405	156.9	280.8	487.3

K^+	mg/L	5.2	4.35	3.8	47.47	0.9	48.37	3.39	4.35	6.07
NO_3^-	mg/L	10.54	4.3	23.22	310.98	0.02	311	1.24	4.3	9.24
NO_2^-	mg/L	0.33	0.001	1.47	16.199	0.001	16.2	0.001	0.001	0.008
pH	mg/L	7.92	7.85	0.33	2.11	7.15	9.26	7.71	7.85	8.04
SO_4^{2-}	mg/L	349.77	268	316.48	2493.5	0.5	2494	165.2	268	447.1
TDS	mg/L	1844.62	1294	1969.5	22060.6	321.4	22382	868.8	1294	2046
Cl^-	mg/L	573.12	269.4	1023.03	11772.08	26.92	11799	144.7	269.4	564.6
Ca^{2+}	mg/L	95.14	79.77	74.59	878.03	4.27	882.3	52.2	79.77	119.7
Br^-	mg/L	0.49	0.2	1.28	13.95	0.05	14	0.05	0.2	0.48

Table S2. The information of the groundwater samples.

stationID	pH	well depth(m)	T(℃)	stationID	pH	well depth(m)	T(℃)	stationID	pH	well depth(m)	T(℃)	stationID	pH	well depth(m)	T(℃)
A001	7.76	100	10.6	A113	7.64	12	9.84	A224	7.36	8	11.3	A336	7.46	12	12.1
A002	7.49	10	12.8	A114	7.62	15	10.6	A225	8.05	20	10.3	A337	7.59	8	11
A003	7.72	17	9.25	A115	7.78	20	10.5	A226	7.65	15	9.99	A338	7.78	15	15.5
A004	7.61	10	9.85	A116	7.64	20	9.95	A227	7.69	20	9.2	A339	7.6	6	11
A005	7.78	15	9.07	A117	7.8	60	10.2	A228	7.7	12	10.2	A340	7.83	8	11.4
A006	8.09	15	9.7	A118	7.82	16	9.17	A229	7.67	12	9.45	A341	7.56	15	15.6
A007	8.23	45	9.98	A119	7.8	13	9.45	A230	7.65	12	10.2	A342	8.63	5	12.7
A008	7.93	18	9.76	A120	7.66	15	10.4	A231	7.64	10	10.3	A343	7.58	7	12.6
A009	7.85	22	9.95	A121	7.79	8	10.3	A232	7.65	12	9.74	A344	7.62	5	12.8
A010	9.05	26	9.73	A122	7.76	15	10.1	A233	7.75	12	9.34	A345	7.86	3	15.1
A011	8.7	10	10.1	A123	7.81	15	10	A234	7.61	15	12.2	A346	7.74	5	13.5
A012	7.62	8	10	A124	7.97	20	10.4	A235	7.54	18	10.1	A347	7.76	6	12.3
A013	7.78	16	9.35	A125	7.72	13	9.4	A236	7.91	15	10.3	A348	8.03	10	11.6
A014	8.89	10	8.99	A126	7.56	31	9.66	A237	7.83	18	10	A349	7.8	7	9.61
A015	7.92	30	9.22	A127	8.08	25	9.68	A238	7.9	10	13	A350	8.43	6	11.3
A016	8.57	23	9.31	A128	8.6	30	9.89	A239	7.75	12	9.84	A351	7.78	11	11.4
A017	7.65	19	9.44	A129	7.87	14	10.3	A240	7.65	12	10.6	A352	7.5	8	11.3
A018	7.92	17	9.01	A130	7.51	3	10.1	A241	7.97	10	9.97	A353	7.68	9	15.2
A019	7.88	10	9.01	A131	7.85	50	9.58	A242	8.31	10	10.6	A354	7.64	8	17
A020	7.94	20	8.78	A132	7.78	8	12	A243	7.69	11	9.61	A355	8.4	5	16.4
A021	7.73	12	9.77	A133	7.6	16	9.71	A244	7.76	12	0	A356	7.72	5	17.1
A022	8.56	5	13.3	A134	7.64	30	10.3	A245	7.72	12	9.72	A357	7.94	12	13.1
A023	7.99	30	10	A135	7.74	80	11	A246	8.13	10	11.5	A358	7.6	4	16.1
A024	8.63	30	10.7	A136	7.75	14	10.3	A247	7.49	12	12	A359	7.85	23	14.2
A025	7.86	100	10.7	A137	8.46	13	9.45	A248	8.05	11	10.3	A360	7.55	18	15.7
A026	8.13	17	9.94	A138	7.92	15	9.39	A249	7.8	11	11.3	A361	7.59	12	10.9
A027	7.74	16	11.2	A139	7.9	13	10.1	A250	8.48	12	9.98	A362	7.54	6	14.1
A028	7.89	24	9.71	A140	8.1	16	9.77	A251	7.75	12	11.7	A363	7.66	80	11.8
A029	8.68	30	9.79	A141	7.88	8	10.1	A252	7.54	16	11.2	A364	7.54	3	18.4

A030	8.28	30	9.48	A142	7.75	16	9.65	A253	7.82	7	11	A365	7.71	14	15.1
A031	7.82	70	12.8	A143	7.66	120	10.2	A254	7.76	13	11.4	A366	7.8	10	11.8
A032	7.87	14	9.17	A144	7.98	24	9.31	A255	8.06	15	9.79	A367	7.63	8	12
A033	7.93	15	9.78	A145	7.6	18	11.5	A256	8.58	12	10.9	A368	7.84	8	11.7
A034	8.09	15	10.4	A146	7.61	11	10.4	A257	7.87	14	10.2	A369	7.8	8	11.4
A035	8.31	22	8.65	A147	7.65	20	11.4	A258	7.67	8	11	A370	8.5	85	13.5
A036	7.89	7	11.9	A148	7.8	13	10.6	A259	7.93	11	11.7	A371	7.71	12	11.6
A037	8.17	30	10.1	A149	7.8	13	10.5	A260	8.09	12	9.35	A372	7.58	7	10.7
A038	7.78	30	9.35	A150	7.65	17	10.5	A261	7.48	10	11.7	A373	7.69	8	10.8
A039	8.01	30	9.85	A151	7.75	12	10.2	A262	7.87	10	10.9	A374	8.56	8	10.8
A040	7.82	24	9.68	A152	7.76	13	9.91	A263	7.77	11	9.95	A375	7.55	15	11
A041	8.97	14	9.37	A153	7.8	15	12.1	A264	7.52	14	11.2	A376	7.66	7	10.8
A042	7.61	12	11.3	A154	7.59	12	10.2	A265	7.73	12	9.81	A377	7.86	9	10.4
A043	7.52	12	9.56	A155	7.6	14	9.95	A266	7.5	10	12.5	A378	7.75	12	11.1
A044	7.75	12	9.18	A156	7.74	15	10.9	A267	8.06	7	10.9	A379	7.6	8	11.1
A045	7.7	30	9.62	A157	7.85	15	10.6	A268	7.86	30	12.4	A380	7.87	8	10.5
A046	7.88	15	9.87	A158	7.68	8	9.77	A269	7.72	12	12.1	A381	7.89	12	11.3
A047	7.81	20	10.5	A159	7.67	13	11.5	A270	7.56	87	11.5	A382	7.88	5	12.1
A048	8.27	25	9.22	A160	7.58	15	9.68	A271	7.94	20	11.8	A383	7.73	7	12
A049	7.63	10	9.7	A161	7.68	14	9.86	A272	8.5	13	14.6	A384	7.63	8	10.6
A050	7.99	8	9.73	A162	7.82	12	11.4	A273	8.01	20	11.4	A385	7.73	7	13.1
A051	7.66	8	9.85	A163	7.81	11	9.83	A274	7.78	21	11.2	A386	7.9	31	12.3
A052	7.66	12	10.1	A164	7.76	9	10.2	A275	7.75	60	10.3	A387	7.82	75	9.11
A053	7.65	14	9.99	A165	7.71	12	11.3	A276	7.91	9	11	A388	7.73	60	9.11
A054	7.78	15	10.2	A166	7.33	15	10.5	A277	7.66	10	11.4	A389	7.8	10	9.21
A055	8.2	13	9.53	A167	7.83	12	9.95	A278	7.96	9	11.8	A390	7.92	10	12
A056	7.83	17	10.3	A168	7.78	12	9.98	A279	7.78	15	11.6	A391	7.77	12	9.51
A057	7.76	14	9.79	A169	7.71	13	10.4	A280	7.62	20	12.7	A392	7.72	12	8.99
A058	7.96	20	9.56	A170	7.73	16	10.5	A281	7.88	10	13.4	A393	7.93	12	8.39
A059	7.98	20	9.91	A171	8.02	15	10.9	A282	7.6	50	11.6	A394	7.76	25	8.43
A060	7.82	15	10.2	A172	8.67	15	10.5	A283	7.61	70	11.7	A395	7.65	20	10.4
A061	7.7	10	10.3	A173	7.92	110	12.9	A284	7.9	70	12.3	A396	7.65	16	10.6
A062	8.48	22	10.6	A174	7.95	60	12.4	A285	8	20	11.1	A397	7.51	18	11.8
A063	8.52	27	9.79	A175	8.8	90	12	A286	7.97	80	11	A398	7.87	28	9.92
A064	8.12	30	9.74	A176	8.81	39	11.5	A287	7.75	12	11.7	A399	7.81	13	10.3
A065	8.19	80	10.2	A177	7.6	12	10.1	A288	7.88	70	10.8	A400	7.7	17	9.89
A066	7.57	15	9.57	A178	8.93	17	10.2	A289	7.93	20	11.1	A401	7.92	28	9.7
A067	7.64	15	10.2	A179	9.13	20	10.3	A290	8.57	15	11.3	A402	7.88	18	10.2
A068	8.45	20	9.54	A180	7.91	15	10.3	A291	8.56	17	10.2	A403	7.89	16	9.95
A069	8.28	20	9.52	A181	9.03	12	10.9	A292	7.87	15	10.6	A404	7.88	24	9.91
A070	8.47	15	9.99	A182	8.9	23	10.6	A293	7.85	10	10.4	A405	7.63	30	11.9
A071	8.48	20	10	A183	8.14	8	10.9	A294	7.6	65	13.5	A406	7.71	14	9.54

A072	8.53	12	9.9	A184	8.72	8	9.62	A295	7.62	60	13.2	A407	7.64	18	9.87
A073	8.51	15	10.1	A185	8.61	22	9.61	A296	7.56	65	12.3	A408	7.82	60	10.2
A074	7.93	30	10.1	A186	7.8	12	9.58	A297	7.78	22	12.5	A409	7.75	20	9.84
A075	7.78	12	11	A187	7.64	13	10.2	A298	7.74	20	11.1	A410	7.62	22	9.78
A076	7.61	30	11	A188	7.78	13	10.9	A299	7.75	70	11.7	A411	7.97	13	10.2
A077	7.79	10	10.7	A189	7.79	100	9.75	A300	7.89	6	10.7	A412	7.75	18	9.74
A078	8.16	18	9.76	A190	7.68	23	10.5	A301	8.1	6	11.9	A413	7.7	15	11.4
A079	8.08	20	9.7	A191	8.75	12	10.6	A302	7.87	84	10	A414	7.7	26	11.5
A080	7.83	17	9.7	A192	7.81	70	11.3	A303	8.18	8	10.3	A415	7.68	15	8.76
A081	8.62	20	9.13	A193	7.84	110	9.06	A304	7.95	10	11.6	A416	7.69	15	8.82
A082	8.43	23	10	A194	7.38	100	14.1	A305	7.69	9	10.5	A417	7.69	12	10.3
A083	8.13	18	9.82	A195	8.45	100	11.6	A306	7.82	10	9.97	A418	8.03	7	9.36
A084	8.29	10	10.2	A196	7.61	11	9.95	A307	7.86	12	9.83	A419	7.61	12	10.5
A085	7.86	16	10.5	A197	7.73	10	13	A308	7.89	12	11.2	A420	7.91	10	9.46
A086	7.91	8	9.63	A198	8.57	15	9.18	A309	8.04	10	10	A421	7.81	15	8.38
A087	9.08	12	10.1	A199	7.63	15	10.5	A310	8.07	14	11.3	A422	7.63	12	10.2
A088	8.33	23	9.84	A200	8.04	8	9.44	A311	7.85	60	10.1	A423	7.72	15	9.84
A089	8.15	4	10.2	A201	7.48	30	11.7	A312	8.05	17	10.7	A424	7.96	20	10.8
A090	7.64	14	10.6	A202	8.19	50	13.1	A313	7.76	18	9.78	A425	8.12	20	11.2
A091	7.65	19	9.8	A203	7.78	70	12.1	A314	7.67	20	10.1	A426	8.02	18	11.5
A092	7.61	13	9.66	A204	8.36	80	13	A315	7.85	88	11.4	A427	7.98	26	10.6
A093	7.72	16	9.37	A205	8.1	38	13.8	A316	7.63	7	10	A428	7.87	17	10.4
A094	8.1	18	10	A206	7.89	40	10.9	A317	7.78	7	10.1	A429	8	14	9.86
A095	7.62	8	10.4	A207	7.96	60	11.2	A318	7.95	16	9.75	A430	7.84	17	10.3
A096	7.89	9	9.4	A208	8.26	30	10.2	A319	7.73	13	11	A431	8.24	15	10.9
A097	7.84	10	10.6	A209	8.1	15	10.3	A320	8.07	12	11.1	A432	8.19	20	10.5
A098	8.24	24	10.8	A210	7.98	15	11.5	A321	7.99	16	10.1	A433	7.98	12	11.1
A099	7.86	15	9.18	A211	8.27	15	9.87	A322	7.79	14	10.6	A434	8.17	16	10.9
A100	7.94	30	9.04	A212	7.89	20	10.7	A323	7.66	14	10.5	A435	7.96	18	10
A101	8.03	12	9.68	A213	8.24	12	10.4	A324	7.69	20	12.2	A436	8.34	18	9.82
A102	8.96	24	9.48	A214	8.04	4	9.15	A325	7.97	28	10.8	A437	7.89	27	10.2
A103	9.1	20	10.2	A215	7.43	8	15	A326	9.26	34	10.4	A438	7.87	43	10.2
A104	7.86	100	9.8	A216	7.67	9	10.1	A327	7.9	80	11.7	A439	7.9	28	11.8
A105	7.69	80	10.4	A217	7.77	10	11.8	A328	7.15	67	11.9	A440	7.93	18	10.5
A106	7.82	13	9.43	A218	7.96	18	13.7	A329	7.96	12	12.7	A441	8.33	16	11.6
A107	7.82	20	10.3	A219	8.1	9	11.6	A330	7.68	40	10.2	A442	8.18	23	11.2
A108	7.67	45	9.84	A220	7.76	12	10.8	A331	7.65	8	10.7	A443	7.67	20	10.9
A109	7.91	50	11.5	A221	7.73	12	11.2	A332	7.99	18	10.1	A444	8.36	18	11.1
A110	7.64	30	12.2	A222	7.58	8	9.64	A333	7.82	2	11.1	A445	8.47	13	11.9
A111	7.85	12	9.71	A223	8.06	8	10.2	A334	8.5	8	10	A446	8.3	15	10
A112	7.77	12	10.7	A224	7.36	8	11.3	A335	7.48	6	11.6	A447	7.92	30	9.72
